# On the Use of Class D Switching-Mode Power Amplifiers in Visible Light Communication Transmitters

**DOI:** 10.3390/s22134858

**Published:** 2022-06-27

**Authors:** Juan R. García-Meré, Juan Rodríguez, Diego G. Lamar, Javier Sebastián

**Affiliations:** Power Supply Group, Electrical Engineering Department, University of Oviedo, 33204 Gijón, Spain; rodriguezmjuan@uniovi.es (J.R.); gonzalezdiego@uniovi.es (D.G.L.); sebas@uniovi.es (J.S.)

**Keywords:** Visible Light Communication (VLC), High-Brightness LEDs (HB-LEDs), Switching-Mode Power Amplifiers (SMPA)

## Abstract

Visible Light Communication (VLC) is a wireless communication technology that uses visible light to transmit information. The most extended implementation of a VLC transmitter employs a DC-DC power converter that biases the High-Brightness LEDs (HB-LEDs), and a Linear Power Amplifier (LPA) that reproduces the communication signal. Unfortunately, the power efficiency of LPAs is very low, thus reducing the overall system efficiency and requiring huge cooling systems to extract the heat. In this work, the use of Class D Switching-Mode Power Amplifiers (SMPAs) is explored in order to overcome that limitation. It is important to note that this SMPA is widely used for different applications, such as audio and RF power amplifiers. Therefore, there are a lot of versions of a Class D SMPA depending on the topology used for the implementation and the modulation strategy used to control the switches. Hence, this work aims to identify, adapt and explain in detail the best approach for implementing a Class D SMPA for VLC. In order to validate the proposed idea, a power-efficient VLC transmitter intended for short-range and low-speed applications was built and evaluated.

## 1. Introduction

Visible Light Communication (VLC) is as a wireless communication system able to alleviate the saturation of the RF spectrum [1,2,3,4]. This technology takes advantage of the wide and unlicensed visible light spectrum (430–750 THz) to transmit information. One of the main advantages of VLC is that it can be implemented by slightly modifying the existing solid-state lighting infrastructure, thus facilitating the technology deployment.

In VLC, the transmission information is performed by modulating the current that flows through High-Brightness LEDs (HB-LEDs), which translate the current modulation into light intensity modulation. In particular, the light intensity emitted by the HB-LEDs [*s*(*t*)] is made up of a DC component (*S_dc_*) and an AC component [*s_ac_*(*t*)] that fulfill the lighting and the communication tasks, respectively.

The VLC driver is a key element of VLC systems because it is in charge of supplying the HB-LEDs properly to obtain the desired *s*(*t*). In order to achieve that target, the output voltage of the VLC driver [*v_o_*(*t*)] is also made up of a DC component (*V_o-dc_*) and an AC component [*v_o-ac_*(*t*)] that determine *S_dc_* and *s_ac_*(*t*), respectively. It is important to note that although conventional HB-LED drivers for lighting applications are able to reach high power efficiency [5], the communication functionality of VLC drivers adds extra power losses, thus reducing the overall system efficiency [6]. The reason is that, in contrast to conventional HB-LED drivers for lighting applications, a VLC driver has to provide not only high-power efficiency, but also high bandwidth and linearity to fulfill the transmission capability [7]. As a consequence, a lot of research efforts during the last years have been made to alleviate that trade-off [8].

**Figure 1:**
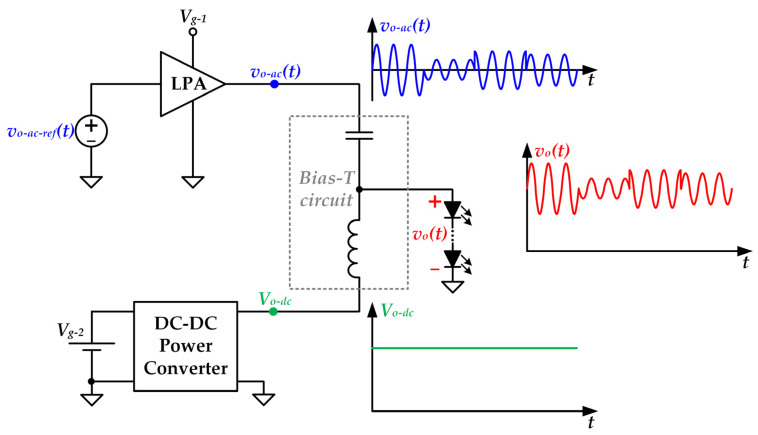
VLC driver based on the use of a DC-DC power converter, a LPA and a bias-T circuit.

The most popular approach for implementing a VLC driver is shown in Figure 1. It is made up of a DC-DC power converter, a Linear Power Amplifier (LPA) and bias-T circuit that are responsible for providing *V_o-dc_*, *v_o-ac_*(*t*) and summing them. This approach is widely used due to its simplicity in terms of control and hardware complexity. Moreover, the LPA provides the high linearity and bandwidth that is required for reproducing passband modulation schemes satisfactorily. It is important to note that these modulation schemes, such as such as dc-biased Quadrature Amplitude Modulation (QAM) and dc-biased Orthogonal Frequency Division Multiplexing (OFDM), are the preferred ones for VLC applications due to their high spectral efficiency and high robustness against the multipath issue of wireless-transmission scenarios [9,10]. Unfortunately, LPAs suffer from low power efficiency (in the range of 10–40%), which leads to high power losses and to the use of huge cooling systems (i.e., heat sink, fan, etc.) to extract the heat [11,12,13].

In contrast to LPAs, Switching-Mode Power Amplifiers (SMPAs) reach high power efficiency (the theoretical power efficiency is 100%) because the employed transistors operate as electronic switches instead of operating in linear mode. Therefore, the LPA of the VLC driver shown in Figure 1 could be replaced by a SMPA in order to alleviate the power efficiency problem. In this sense, previous works explored the use of Class E SMPAs [14,15]. Unfortunately, that SMPA class is not able to modulate the amplitude of the communication carrier and, consequently, that approach only allows us to reproduce phase-modulated schemes. Moreover, the Class E SMPA suffers from high voltage stresses across the switches and low slew-rates. In this sense, the use of a LPA that linearly assists the Class E SMPA was proposed to increase the slew-rate [14]. However, that LPA also damages the power efficiency and increases the complexity of the VLC driver. Furthermore, it does not solve the limitation related to the reproduction of amplitude-modulated schemes. Alternatively, two VLC drivers based on the use of Class E SMPAs can be combined to reproduce amplitude-modulated schemes by implementing the outphasing technique [15]. Unfortunately, that approach leads to high complexity in terms of both hardware and control, which are critical parameters to be consider for designing HB-LED bulbs [16].

Another approach for implementing a VLC driver able to reach high power efficiency consists in using a fast-response DC-DC converter that not only biases the HB-LEDs by providing *V_o-dc_*, but also reproduces the communication signal by generating *v_o-ac_*(*t*) [17,18,19,20,21,22,23,24,25,26]. However, that approach also leads to a noticeable increase in both hardware and control complexity in comparison to the approach shown in Figure 1, especially when the VLC driver must be able to reproduce passband modulation schemes [23,24,25,26].

In this work, a VLC driver based on replacing the LPA with a SMPA is proposed to fulfill the following objectives:

- Alleviate the power efficiency problem of the approach based on the use of a LPA;
- Preserve the simplicity in terms of hardware and control of the approach based on the use of a LPA;
- Preserve the capability for reproducing any kind of passband modulation scheme.

In order to fulfill the aforementioned objectives, the use of a Class D SMPA is proposed, taking into account the benefits reported in different applications, such as audio and RF power amplifiers. In this way, this work aims to explore the different implementations that are proposed for Class D SMPAs, to identify the best approach for VLC applications and to adapt it.

The paper is organized as follows. Section 2 is focused on exploring different implementations of Class D SMPAs. The best approach for implementing a VLC transmitter is explained in Section 3. The experimental results are given in Section 4. Finally, the conclusions are gathered in Section 5.

## 2. Identifying the Best Class D SMPA Implementation for VLC

### 2.1. General Description of a Class D SMPA

**Figure 2:**
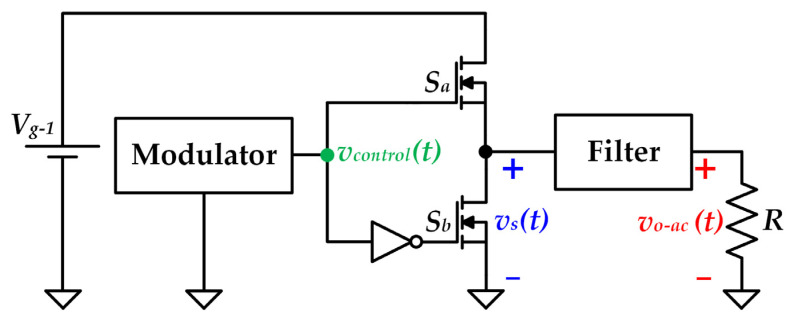
General scheme of a Class D SMPA.

The Class D amplifier is one of the fundamental SMPAs. Although there are several topologies that could be used for implementing this SMPA class [27,28], the present work is focused on the approach shown in Figure 2. Other implementations increase the complexity in terms of hardware by requiring additional voltage sources or transformers. In contrast, the topology selected in this work for implementing the Class D SMPA provides the highest hardware simplicity, which is an essential requirement of HB-LED bulbs [16].

As can be seen, the topology is supplied by a DC voltage source (*V*_*g*−1_). Moreover, a resistive load (*R*) is connected to the output port. The power stage of the Class D SMPA is made up of a non-dissipative filter and two complementary controlled switches (*S_a_* and *S_b_*) that are implemented with Metal-Oxide-Semiconductor Field-Effect Transistors (MOSFETs).

The operating principle of the Class D SMPA consists in generating a constant-frequency train of voltage pulses [*v_s_*(*t*)] with an amplitude equal to *V*_*g*−1_. After that, the undesired harmonics of *v_s_*(*t*) are removed by the filter in order to provide the desired communication signal at the output [i.e., *v_o-ac_*(*t*)]. It is important to note that the pulses pattern of *v_s_*(*t*) is controlled by the modulator by means of the control signal that feeds the MOSFETs gate [*v_control_*(*t*)]. Another important point is that the filter design must be faced taking into account the modulation strategy used to control the Class D SMPA (further details will be given in the following sections).

**Figure 3:**
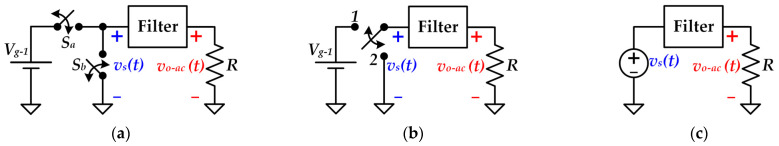
Equivalent circuits of a Class D SMPA: (**a**) Considering two complementary controlled switches. (**b**) Considering a single-pole double-throw switch. (**c**) Considering a pulse-voltage source at the input of the filter.

In order to understand the generation of *v_s_*(*t*), the two possible states of the two complementary controlled switches will we briefly explained. During state 1 [i.e., when *v_control_*(*t*) is in high state], MOSFET *S_a_* is activated and MOSFET *S_b_* is deactivated, thus operating as a short-circuit and as an open circuit, respectively. As a consequence, the voltage applied at the input of the filter [i.e., *v_s_*(*t*)] is equal to *V*_*g*−1_. Furthermore, during state 2 [i.e., when *v_control_*(*t*) is in low state], MOSFET *S_a_* is deactivated and MOSFET *S_b_* is activated. As a result, MOSFET *S_a_* and MOSFET *S_b_* operate as an open circuit and as a short-circuit, respectively. In this case, *v_s_*(*t*) is equal to 0V. In summary, the two MOSFETs can be seen as two single-pole single–throw switches (see Figure 3a). Moreover, since both MOSFETs are controlled complementarily, they can be replaced by a single-pole double-throw switch (see Figure 3b) to further simplify the operation description. The Class D SMPA switches between states 1 and 2, thus generating the pulse-pattern determined by the modulator. Therefore, the equivalent circuit shown in Figure 3c can be used to represent its operation as a pulse-voltage source of amplitude *V*_*g*−1_ that is filtered to remove the undesired harmonics.

### 2.2. Pulse-Width Modulated Class D SMPA

**Figure 4:**
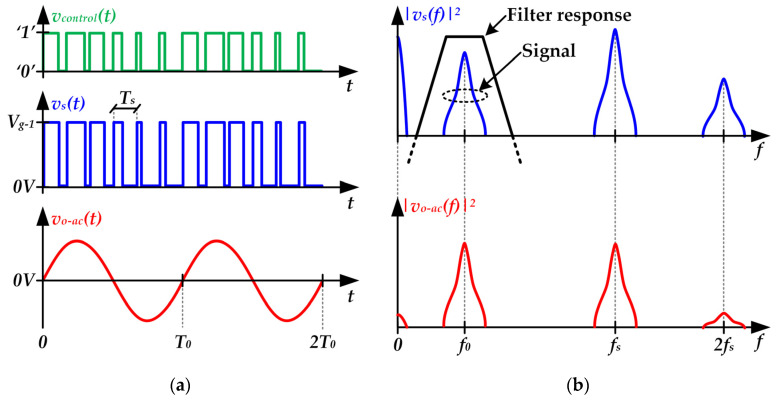
Main voltage waveforms of a PWM Class D SMPA: (**a**) Time domain. (**b**) Frequency domain. Note that *T_s_* and *T*_0_ are the switching period (i.e., *1/f_s_*) and the communication carrier period (i.e., *1/f*_0_), respectively.

Pulse-Width Modulation (PWM) is widely used to control Class D SMPAs in applications such as audio amplifiers [29,30,31]. Figure 4 shows the main voltage waveforms necessary to understand its operating principle.

As can be seen, the modulation strategy consists in generating a pulse-pattern with constant-frequency where the pulse width determines the output voltage (see Figure 4a). After that, the undesired harmonics are removed by the filter (see Figure 4b). In this modulation strategy, the frequency of the pulse-voltage waveform (*f_s_*), which is called switching frequency, must be much higher than the frequency of the sinusoidal signal that is used as communication carrier (i.e., *f*_0_) in order to achieve enough rejection of the undesired harmonics (typically, *f_s_* > *10·f*_0_).

At this point, it is important to note that although SMPAs are implemented with non-dissipative filters and that the MOSFETs operate as electronic switches, the theoretical 100% power efficiency is never reached. In the case of PWM Class D SMPAs for VLC applications, the switching losses of the MOSFETs would be the most critical source of power loss. The key point is that the transition between the two possible states of a MOSFET when it operates as an electronic switch is not instantaneous. During these transitions, the MOSFET dissipates power because it withstands voltage and drives current at the same time. Therefore, the MOSFET consumes power every time it changes its state and, consequently, switching losses rise with *f_s_*. In the case of using a PMW Class D SMPA for VLC, it would require a *f_s_* of tens of MHz, taking into account that *f*_0_ can reach the MHz range (the HB-LED bandwidth ranges between 3 and 20 MHz depending on the device kind [9]). However, reaching that switching frequency is not straightforward because it will lead not only to remarkable switching losses, but also to a complex design from the hardware and control perspective.

### 2.3. Radiofrequency Pulse-Width Modulated Class D SMPA

The Radiofrequency Pulse-Width Modulation (RF-PWM) technique was introduced by Raab in 1973 [32], demonstrating its capability to reproduce amplitude-modulated signals with a carrier of limited frequency (around 100 kHz). One of the main benefits of this technique is that the required switching frequency is equal to the frequency of the communication carrier (i.e., *f_s_* = *f*_0_), thus alleviating the switching losses problem of the conventional PWM technique. Unfortunately, the low speed of the analog circuitry required for implementing the modulator prevented its use at the MHz range for 25 years [33]. The development of digital control technology has avoided the bottleneck caused by the analog-based modulator, thus promoting its use even at the GHz range for implementing RF SMPAs [34,35,36,37]. In VLC, the frequency is not as challenging as in that case. This fact enables the use of general-purpose digital platforms, which is critical for accomplishing the simplicity requirement of HB-LED bulbs.

**Figure 5:**
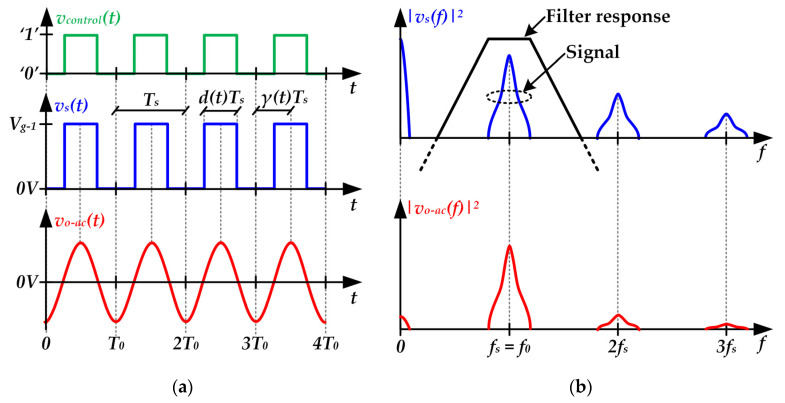
Main voltage waveforms of a RF-PWM Class D SMPA under steady-state conditions [i.e., considering fixed values of *d*(*t*) and *γ*(*t*)]: (**a**) Time domain. (**b**) Frequency domain.

Figure 5 shows the main voltage waveforms necessary to understand the operation of a RF-PWM Class D SMPA. In this case, the filter passes the fundamental harmonic of *v_s_*(*t*) (i.e., the harmonic at *f_s_*), which is used as communication carrier (see Figure 5b). Moreover, the technique is based on controlling not only the pulse width, but also the pulse position in order to perform the amplitude and phase modulation of the communication carrier, respectively. In particular, the dimensionless control parameters *d*(*t*) and *γ*(*t*) are used to control the pulse width and the pulse position, respectively (see Figure 5a). It is important to note that *d*(*t*) ranges between 0 and 0.5, and *γ*(*t*) ranges between 0 and 1.

The Fourier analysis allows us to express the pulse-voltage waveform at the input of the filter as follows:

(1) $$v_{s}\left(t\right)=\overset{\infty }{\underset{k=1}{\sum }}\frac{2V_{g-1}}{k\pi }\sin\left[k\pi d\left(t\right)\right]\cos\left[k2\pi f_{s}t-k2\pi \gamma \left(t\right)\right].$$

Taking into account that the filter only passes the component at *f_s_*, the output voltage is equal to the first harmonic of *v_s_*(*t*) and, consequently, can be expressed as follows:

(2) $$v_{o-ac}\left(t\right)=\frac{2V_{g-1}}{\pi }\sin\left[\pi d\left(t\right)\right]\cos\left[2\pi f_{s}t-2\pi \gamma \left(t\right)\right].$$

The amplitude modulation term of Equation (2) is:

(3) $$A_{v}\left(t\right)=\frac{2V_{g-1}}{\pi }\sin\left[\pi d\left(t\right)\right].$$

Furthermore, the phase modulation term of Equation (2) is:

(4) $$\phi_{v}\left(t\right)=-2\pi \gamma \left(t\right).$$

**Figure 6:**
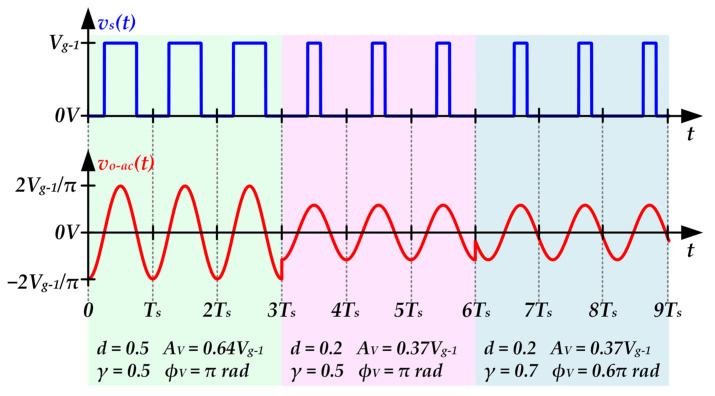
Main voltage waveforms of a RF-PWM Class D SMPA that changes the control parameters over time in order to reproduce a dc-biased QAM scheme.

Figure 6 shows the main voltage waveforms of a RF-PWM Class D SMPA when the communication carrier is modulated according to a dc-biased QAM scheme. As can be seen, the control parameters are changed over time in order to reproduce the communication signal. In that example, three symbols of the dc-biased QAM scheme are transmitted. Note that a symbol period of three carrier periods is considered. The control parameters are modified each symbol period to generate the target output voltage. In *t* = *3T_s_*, *d*(*t*) is reduced to reproduce a symbol with lower amplitude. After that, in *t = 6T_s_*, *γ*(*t*) is changed in order to modify the phase of the third symbol maintaining the amplitude of the second one.

## 3. Adapting a RF-PWM Class D SMPA for VLC

### 3.1. Characterizing the HB-LEDs

**Figure 7:**
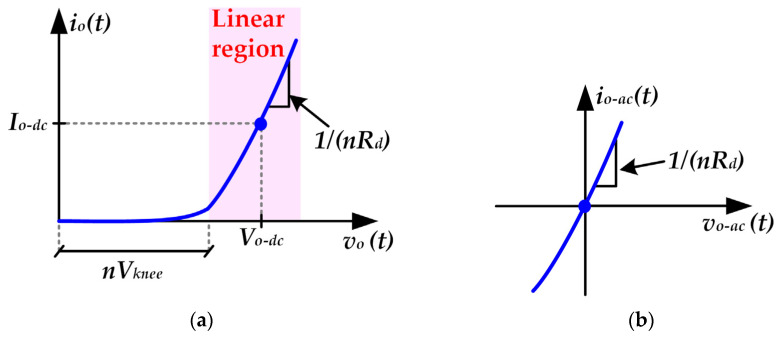
Electrical behavior of a string of *n* HB-LEDs used for VLC: (**a**) Current-voltage curve of a HB-LED. (**b**) Equivalent current-voltage curve seen by the RF-PWM Class D SMPA as load.

Studying the electrical behavior of the HB-LEDs is mandatory to model the load supplied by the RF-PWM Class D SMPA for VLC. The light intensity emitted by the HB-LEDs is proportional to the current that flows through them [*i_o_*(*t*)]. Taking into account that the output of a Class D SMPA operates as a voltage source, modeling the relationship between *i_o_*(*t*) and *v_o_*(*t*) is essential for designing the proposed VLC transmitter. Figure 7a shows the current-voltage plot of a HB-LED string. That relationship can be expressed as follows when the HB-LEDs operate in the linear region:

(5) $$i_{o}\left(t\right)=\frac{1}{nR_{d}}\left[v_{O}\left(t\right)-nV_{knee}\right],$$

where *n*, *R_d_* and *V_knee_* are the number of HB-LEDs, their dynamic resistance and the knee voltage, respectively.

The current-voltage curve shown in Figure 7b models the equivalent load seen by the RF-PWM Class D SMPA taking into account that the DC-DC power converter of the VLC driver is in charge of biasing the HB-LEDs (see Figure 1). In particular, the relationship between the AC output current and the AC output voltage of the VLC driver can be expressed as follows:

(6) $$i_{o-ac}\left(t\right)=\frac{v_{O-ac}\left(t\right)}{nR_{d}}.$$

Therefore, the equivalent load seen by the RF-PWM Class D SMPA has a purely resistive behavior that can be expressed as:

(7) $$R=nR_{d}.$$

### 3.2. Using a Series-Resonant LC Circuit as Output Filter

Although several passband filters could be used for implementing a Class D SMPA [27,28], this work is focused on the series-resonant *LC* circuit due to its simplicity in terms of hardware. The filter must pass the fundamental harmonic of *v_s_*(*t*) and must reject the other ones. In this way, the following expression should be followed in order to choose the inductor *L* and the capacitor *C*:

(8) $$LC=\frac{1}{{\left(2\pi f_{0}\right)}^{2}}.$$

**Figure 8:**
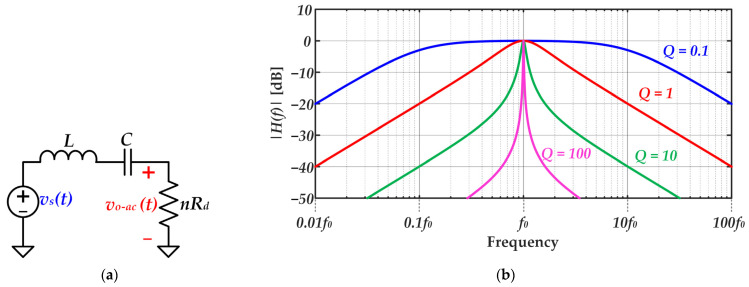
Series-resonant *LC* circuit characterization: (**a**) Equivalent circuit model. (**b**) Filter response for different *Q* values.

There are infinite combinations of *L* and *C* values that satisfy Equation (8). However, each combination leads to a different filter behavior. The transfer function between the output and input voltages of the equivalent circuit shown in Figure 8a can be expressed as follows:

(9) $$H\left(s\right)=\frac{v_{o-ac}\left(s\right)}{v_{s}\left(s\right)}=\frac{CnR_{d}s}{LCs^{2}+nR_{d}Cs+1}.$$

Moreover, the high cutoff frequency (*f_c-h_*) and the low cutoff frequency (*f_c-l_*) are:

(10) $$f_{c-h}=\frac{1}{2\pi }\sqrt{\frac{1}{LC}+\frac{{\left(nR_{d}\right)}^{2}}{2L^{2}}\left(1+\sqrt{1+\frac{4L}{C{\left(nR_{d}\right)}^{2}}}\right)},$$

(11) $$f_{c-l}=\frac{1}{2\pi }\sqrt{\frac{1}{LC}+\frac{{\left(nR_{d}\right)}^{2}}{2L^{2}}\left(1-\sqrt{1+\frac{4L}{C{\left(nR_{d}\right)}^{2}}}\right)}.$$

In order to evaluate the selectivity achieved by the series-resonant *LC* circuit, the quality factor definition is introduced:

(12) $$Q=\frac{Center\;frequency}{Bandwidth}=\frac{f_{0}}{f_{c-h}-f_{c-l}}=\frac{2\pi f_{0}L}{nR_{d}}.$$

**Figure 9:**
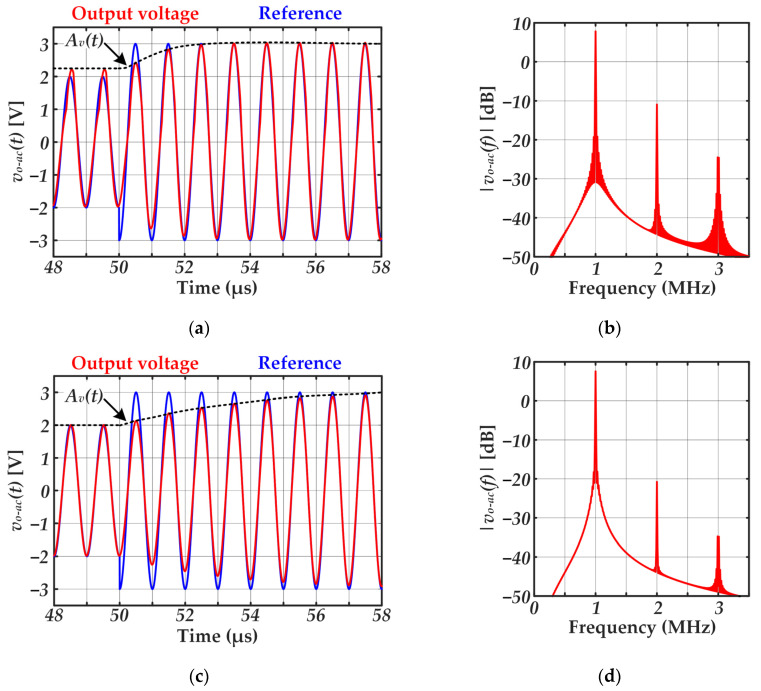
Response to an amplitude step from 2 V to 3 V at *t* = 50 μs considering different *Q* values. Note that for this example, *f*_0_ is 1 MHz and *V*_*g*−1_ is 5 V. In order to track the step of the reference amplitude, a duty cycle step from 0.215 to 0.39 is performed at *t* = 50 μs. (**a**) Time domain results for *Q* = 3. (**b**) Frequency domain results for *Q* = 3. (**c**) Time domain results for *Q* = 10. (**d**) Frequency domain results for *Q* = 10.

According to Equation (12), for given *f*_0_ and *R_d_* values, *Q* rises with *L*. As Figure 8b shows, the higher the *Q* value, the higher the selectivity of the series-resonant *LC* circuit. At this point, the trade-off between selectivity and bandwidth arises. On the one hand, high selectivity is desired because it leads to higher rejection of the undesired harmonics and, consequently, lower distortion of the reproduced signal. On the other hand, the bandwidth falls with the selectivity, thus reducing the bit rate that can be achieved. Figure 9 exemplifies that tradeoff comparing the response to an amplitude change in the communication carrier for different *Q* values. As can be seen, lower *Q* values enable faster changes in the carrier amplitude (i.e., higher bandwidth), which would enable lower symbol periods and, therefore, higher bit rates. However, that is achieved at the expense of reducing the rejection of the undesired harmonics, which is translated into a higher distortion that may jeopardize the communication due to the excessive error.

Another important point that must be taken into account when choosing *L* and *C* values is that the amplitudes of the undesired harmonics change as the pulse width is modulated [see Equation (1)]. In particular, the amplitude modulation of the *k^th^* harmonic of *v_s_*(*t*) can be expressed as follows:

(13) $$A_{v-k}\left(t\right)=\frac{2V_{g-1}}{k\pi }\cdot \left|\sin\left[k\pi d\left(t\right)\right]\right|.$$

**Figure 10:**
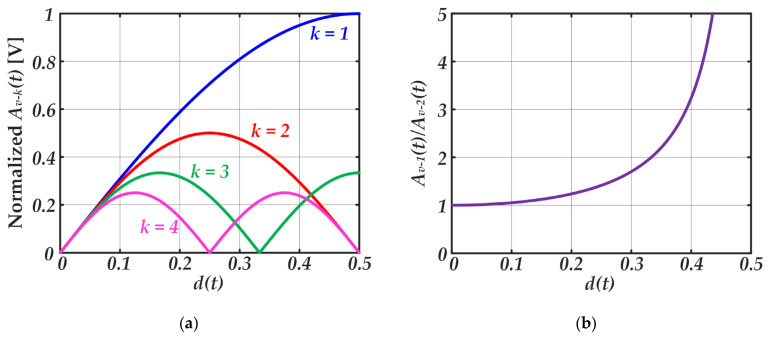
Analysis of the pulse-width modulation impact on the harmonics amplitudes: (**a**) Amplitude modulation of each harmonic versus *d*(*t*). (**b**) Ratio between the amplitudes of the first and the second harmonic.

Figure 10a shows the amplitude modulation of each harmonic versus *d*(*t*). The second harmonic of *v_s_*(*t*) is the most critical harmonic because is the closest one to the communication carrier and, consequently, the one that is least rejected by the filter. Figure 10b shows the ratio between the amplitudes of both harmonics. It can be seen the worst case in terms of distortion appears for low *d*(*t*) values.

As a conclusion, a *Q* value of 5–10 is recommended to address the design of the RF-PWM Class D SMPA for VLC. On the one hand, that *Q* range leads to a selectivity high enough to minimize the distortion caused by the undesired harmonics. On the other hand, it minimizes the penalization in terms of bandwidth, thus maximizing the achievable bit rate.

### 3.3. Control System

**Figure 11:**
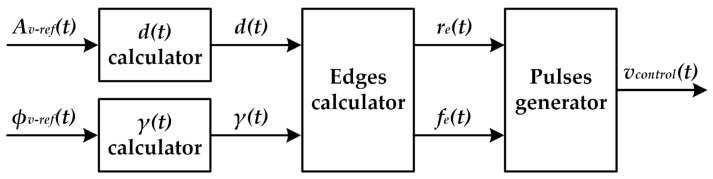
Blocks diagram of the control system used to implement the modulator of the RF-PWM Class D SMPA for VLC.

Figure 11 shows the main blocks necessary to generate the pulse-voltage waveform that controls the MOSFETs [i.e., *v_control_*(*t*)] of a RF-PWM Class D SMPA used for VLC applications. Note that the desired amplitude modulation [*A_v-ref_*(*t*)] and the desired phase modulation [*ϕ_v-ref_*(*t*)] are the system inputs. A brief description of each block can be found below.

#### 3.3.1. *d*(*t*) Calculator

This block calculates the duty cycle value required for performing the amplitude modulation indicated by *A_v-ref_*(*t*). Taking into account the relationship between the amplitude modulation and the duty cycle indicated by Equation (3), the block solves the following equation:

(14) $$d\left(t\right)=\frac{\sin^{-1}\left[\frac{\pi A_{v-ref}\left(t\right)}{2V_{g-1}}\right]}{\pi }.$$

That equation can be easily solved with a look-up table implemented in a general-purpose digital platform.

#### 3.3.2. *γ*(*t*) Calculator

Determining *γ*(*t*) is straightforward taking into account the linear relationship between that control parameter and *ϕ_v_*(*t*). Hence, the equation that has to be solved can be derived from Equation (4), which yields the following expression:

(15) $$\gamma \left(t\right)=-\frac{\phi_{v}\left(t\right)}{2\pi }.$$

#### 3.3.3. Edges Calculator

**Figure 12:**
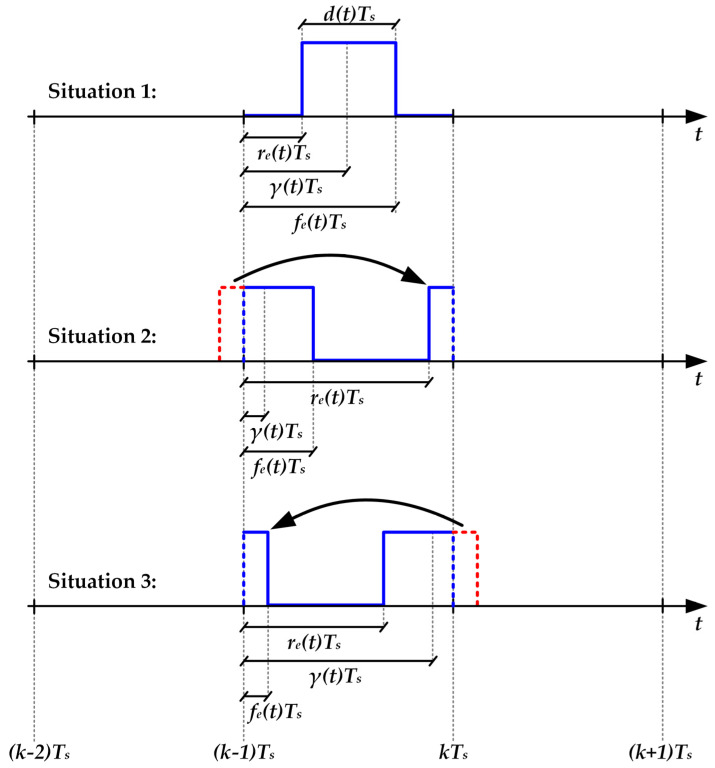
Situations to be considered for generating the voltage pulses of *v_control_*(*t*).

Once *d*(*t*) and *γ*(*t*) are determined, the calculation of the rising edges [*r_e_*(*t*)] and the falling edges [*f_e_*(t)] of *v_control_*(*t*) can be addressed. As Figure 12 shows, three situations can appear depending on the *d*(*t*) and *γ*(*t*) values.

- **Situation 1:** the pulse to be generated does not try to exceed its switching period. This situation appears if the following condition is satisfied:

(16) $$\frac{d\left(t\right)}{2}<\gamma \left(t\right)<1-\frac{d\left(t\right)}{2}.$$

In this case, the rising edge satisfies:

(17) $$r_{e}\left(t\right)=\gamma \left(t\right)-\frac{d\left(t\right)}{2},$$

and, moreover, the falling edge can be determined as follows:

(18) $$f_{e}\left(t\right)=\gamma \left(t\right)+\frac{d\left(t\right)}{2}.$$

- **Situation 2:** the pulse to be generated tries to invade the previous switching period. This situation appears if the following condition is satisfied:

(19) $$\gamma \left(t\right)<\frac{d\left(t\right)}{2}.$$

In this case, the part of the pulse that tries to invade the previous switching period must be moved to the end of the correct one. Therefore, the rising edge can be calculated as follows:

(20) $$r_{e}\left(t\right)=\gamma \left(t\right)-\frac{d\left(t\right)}{2}+1,$$

and, moreover, Equation (18) is valid for determining the falling edge.

- **Situation 3:** the pulse to be generated tries to invade the next switching period. This situation appears if the following condition is satisfied:

(21) $$\gamma \left(t\right)>1-\frac{d\left(t\right)}{2}.$$

In this case, the part of the pulse that tries to invade the next switching period must be moved to the beginning of the correct one. Hence, the rising edge can be determined with Equation (17). Furthermore, the falling edge can be expressed as follows:

(22) $$f_{e}\left(t\right)=\gamma \left(t\right)+\frac{d\left(t\right)}{2}-1.$$

### 3.4. Choke Inductor and Coupling Capacitor of the Bias-T Circuit

In order to complete the adaptation of the RF-PWM Class D SMPA to VLC, the design of the bias-T circuit shown in Figure 1 must be addressed. In our case, only the choke inductor is necessary to be included because the function of the coupling capacitor is fulfilled by the capacitor of the series-resonant *LC* circuit. The objective of the choke inductor is to ensure that the signal provided by RF-PWM Class D SMPA reaches the HB-LED load, thus preventing any leakage to the DC-DC power converter. In this way, the choke inductor must operate as an open circuit for the frequencies of the communication signal. Therefore, the following condition must be satisfied:

(23) $$L_{ch}\gg \frac{nR_{d}}{2\pi f_{s}}.$$

## 4. Experimental Results

### 4.1. Prototype Description

**Figure 13:**
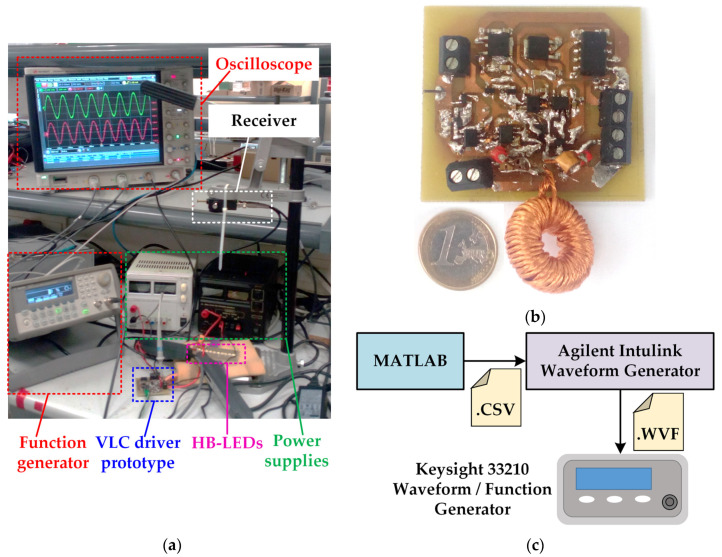
Experimental VLC link: (**a**) Setup. (**b**) RF-PWM Class D SMPA prototype. (**c**) Scheme of the digital platforms used to generate *v_control_*(*t*).

A power-efficient VLC transmitter prototype intended for short-range and low-speed applications was built to validate the proposed idea. Figure 13 shows the setup used to test the VLC link (Figure 13a), the RF-PWM Class D SMPA prototype (Figure 13b) and the scheme of the digital platforms used to generate *v_control_*(*t*) (Figure 13c). As can be seen, the control system was implemented in MATLAB, where the rising and falling edges were calculated. Then, the generated .CSV file was used by Agilent Intulink Waveform Generator software to generate the .WVF file required by Keysight 33,210 Waveform/Function Generator to provide the desired *v_control_*(*t*). It is important to note that this equipment is a general-purpose function generator. Moreover, other general-purpose digital platforms, such as conventional microcontrollers or Field-Programmable Gate Arrays (FPGAs), can be easily programmed in order to implement the proposed control system. PDA10A-EC is used as receiver of the VLC link (see Figure 13a). This device is made up of a photodiode and transimpedance amplifier that provide an output voltage waveform proportional to the received light intensity.

The main components of the RF-PWM Class D SMPA are detailed in Table 1. It is important to note that *V*_*g*−1_ is 6.3 V in order to maximize the use of the HB-LED linear region. Moreover, the HB-LED string is made up of 10 devices connected in series, which lead to an equivalent load of 20 Ω. Taking into account that the bandwidth of the HB-LEDs is around 3 MHz, the selected center frequency of the communication carrier (i.e., *f*_0_) and, consequently, the switching frequency (i.e., *f_s_*) are equal to 1.5 MHz. Furthermore, the *Q* value of the series-resonant LC circuit is set to 10 (*L* = 21.22 μH and *C* = 530.32 pF). Regarding the choke inductor, it was set to 350μH. It is important to note that a power supply that delivers 250 mA is used to bias the HB-LEDs. The implemented RF-PWM Class D SMPA achieves a power efficiency of 77%, thus reaching a remarkable improvement with respect to LPA approaches.

**Figure 14:**
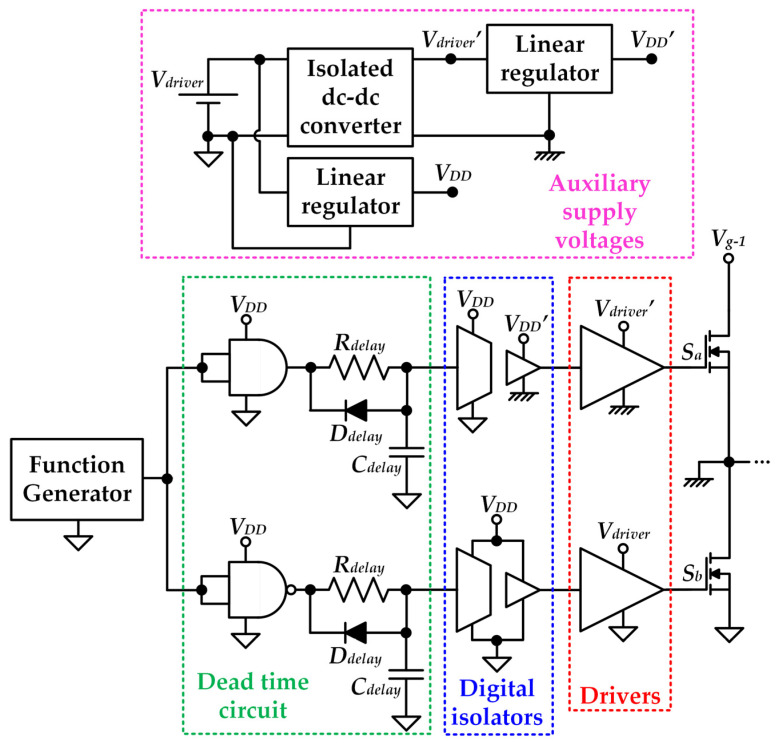
Scheme of the circuit in charge of driving the MOSFETs. Note that a power supply provides *V_driver_* = 9 V.

The circuit used to drive the MOSFETs is shown in Figure 14. This part of the prototype must be carefully designed taking into account that the RF-PWM Class D SMPA operates in the MHz range. Therefore, minimizing the parasitic components of the Printed Circuit Board (PCB) layout is critical. It is important to note that, in principle, MOSFET *S_b_* would not require a digital isolator because its source is connected to ground. However, it is recommended to include it in order to match the time delay introduced by the digital isolator of *S_a_*.

### 4.2. Prototype Tests

**Figure 15:**
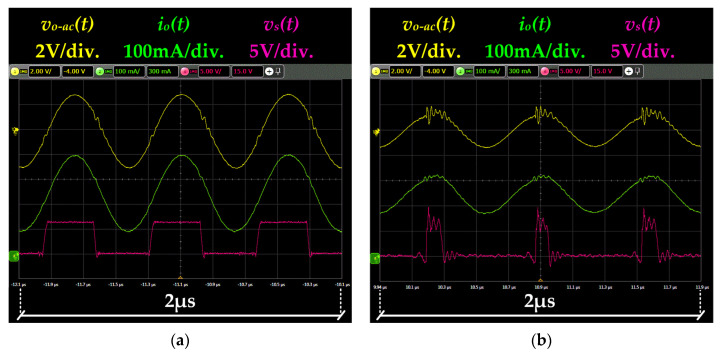
Main voltage and current waveforms under steady-state conditions (**a**) Results for *d*(*t*) = 0.5. (**b**) Results for *d*(*t*) = 0.2.

Two tests were performed prior to validate the VLC link. The first one was focused on evaluating the static operation of the RF-PWM Class D SMPA. In particular, the generated pulse-voltage waveform [i.e., *v_s_*(*t*)], the output voltage [i.e., *v_o-ac_*(*t*)] and the current that flows through the HB-LEDs [i.e., *i_o_*(*t*)] were measured under steady-state conditions for two possible amplitudes of the communication carrier. As Figure 15 shows, the prototype is able to provide an accurate sinusoidal waveform. The results shown in Figure 15b have more distortion than the ones of Figure 15a due to the higher impact of the second harmonic of *v_s_*(*t*) on the output voltage. These results match with the explanation provided in Section 3.2: the ratio between the amplitude of the desired harmonic and that of the second one falls with *d*(*t*), which leads to higher distortion for low amplitudes.

**Figure 16:**
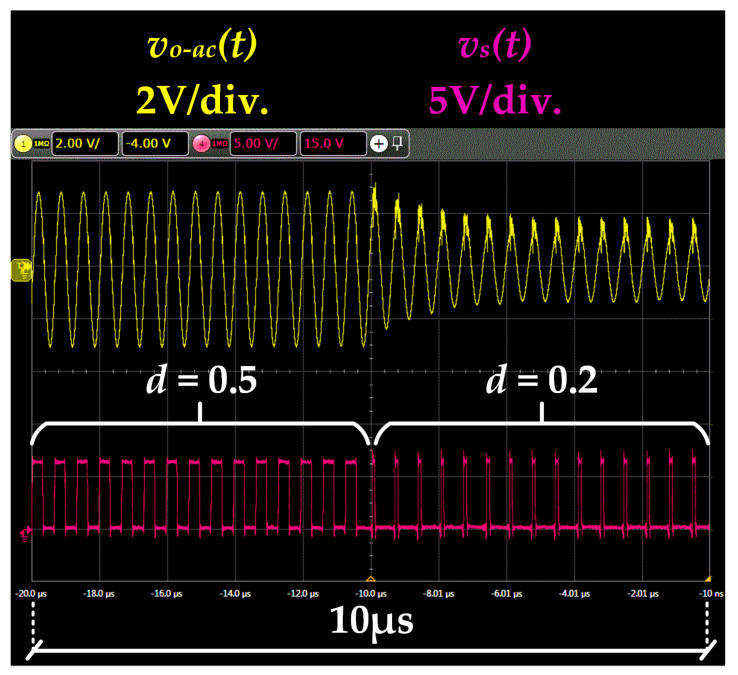
Prototype response to duty cycle change from 0.5 to 0.2.

The second test aims to evaluate the dynamic operation of the RF-PWM Class D SMPA by performing amplitude steps. Figure 16 shows the response of the prototype to a duty cycle change from 0.5 to 0.2. As can be seen, the measurements match with the explanation provided in Section 3.2.

### 4.3. VLC Link Tests

**Figure 17:**
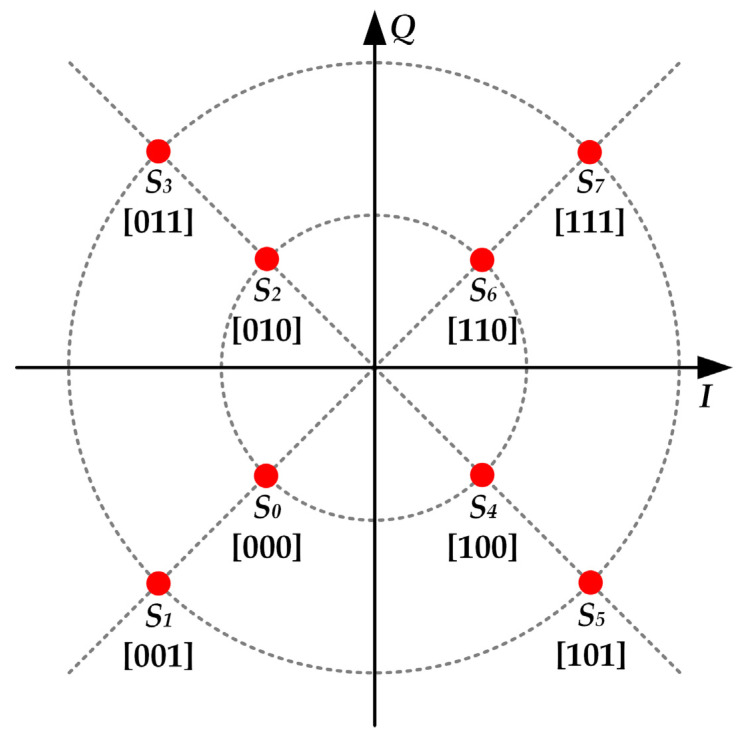
Constellation diagram of the reproduced dc-biased 8-QAM scheme.

The dc-biased 8-QAM scheme shown in Figure 17 was reproduced to demonstrate the communication capability of the prototype. Furthermore, a symbol period of 40 carrier periods was set, which leaded to a bit rate of 112.5 kbps.

**Figure 18:**
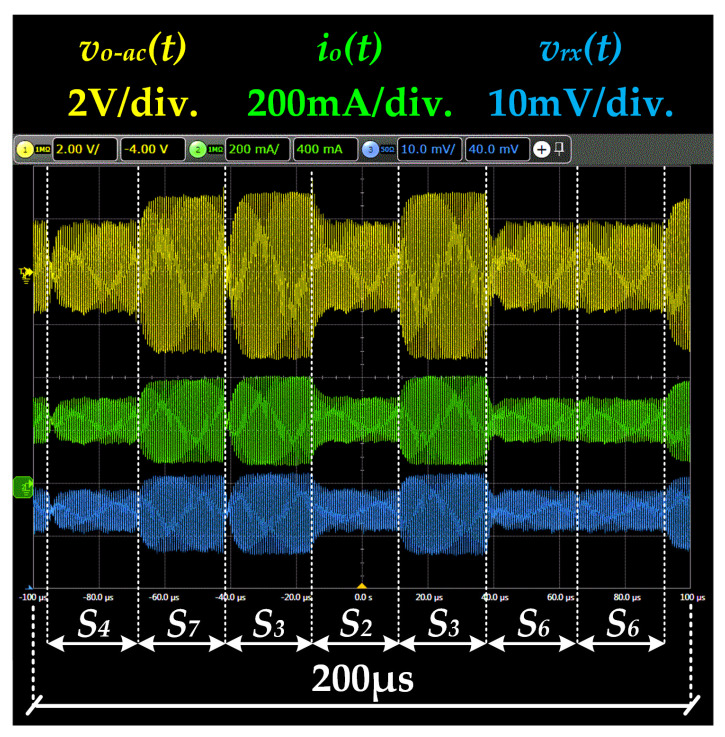
Main waveforms of the VLC link when the dc-biased 8-QAM scheme is reproduced.

Figure 18 shows the main voltage and current waveforms obtained when the VLC link was tested. In particular, the figure shows seven symbols of a longer sequence. It is important to note that *v_rx_*(*t*) is the received signal.

**Figure 19:**
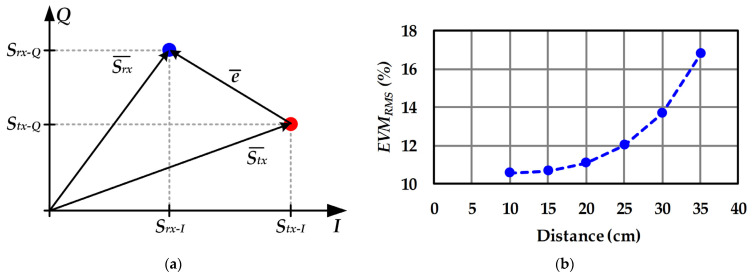
*EVM_RMS_* analysis: (**a**) Graphical interpretation. (**b**) Experimental results versus distance.

To further characterize the VLC system, the root mean square value of the error vector magnitude (*EVM_RMS_*) was evaluated. *EVM_RMS_* is a widely used figure-of-merit based on calculating the error vector for a sequence of *N* symbols, taking into account the average power of the transmitted ones:

(24) $$EVM_{RMS}\left(\%\right)=100\sqrt{\frac{\sum_{i=1}^{N}{\left|\bar{e_{i}}\right|}^{2}}{\sum_{i=1}^{N}{\left|S_{tx-i}\right|}^{2}}}.$$

where $\bar{e_{i}}$ and *S_tx-i_* are the *i^th^* error vector and the *i^th^* transmitted symbol, respectively. It is important to note that $\bar{e_{i}}$ is a complex number that evaluates the difference between the *i^th^* received symbol (*S_rx-i_*) and the *i^th^* ideal transmitted symbol (see Figure 19a):

(25) $$\bar{e_{i}}=S_{rx-i}-S_{tx-i}$$

Figure 19b shows the measured *EVM_RMS_* for different link distances. Typically, *EVM_RMS_* values below 15% are recommended for wireless communication systems [38]. Therefore, the prototype is a suitable option for short-range communications.

## 5. Conclusions

The use of SMPA in VLC transmitters is an interesting approach for alleviating the problems derived from the low power efficiency offered by LPAs. In contrast to other solutions, the proposed RF-PWM Class D SMPA is able to increase the power efficiency, to reproduce any kind of passband modulation scheme and, moreover, to preserve the simplicity of the approach based on the use of a LPA. Furthermore, the adapting exercise that has been performed allows us to identify the key points required for facing the design, the control and the hardware implementation. The prototype reported in the experimental section allows us to validate the idea for power-efficient, short-range, and low-speed VLC applications. More challenging specifications could be explored by increasing the complexity of the modulation scheme, reducing the symbol period and the receiver.

## Figures and Tables

**Table 1:** Main components of the RF-PWM Class D SMPA prototype.

Component	Product Number	Manufacturer
HB-LEDs	XLAMP MX-3 LEDs	Cree
MOSFETs	SSM3K336R	Toshiba
Drivers	EL7156	Renesas
Digital isolators	ISO7220M	Texas Instruments
Isolated dc-dc converter	ISF1212A	XP Power
Linear regulator	MC7805	On Semiconductor
AND gate	SN74LVC1G08	Texas Instruments
NAND gate	SN74LVC1G00	Texas Instruments
Diodes	DB3X313F	Panasonic

## Data Availability

Not applicable.
